# Rare variants in cholesterol transporter genes in patients with lipid metabolism disorders

**DOI:** 10.18699/vjgb-26-53

**Published:** 2026-05

**Authors:** A.D. Izyumchenko, M.N. Grunina, K.V. Dracheva, O.A. Chumakova, K.O. Tanayants, K.V. Legostaeva, A.N. Kulikov, O.A. Berkovich, E.I. Baranova, S.N. Pchelina, V.V. Miroshnikova

**Affiliations:** Pavlov First St. Petersburg State Medical University, St. Petersburg, Russia Petersburg Nuclear Physics Institute named by B.P. Konstantinov of National Research Centre “Kurchatov Institute”, Gatchina, Leningrad region, Russia; Pavlov First St. Petersburg State Medical University, St. Petersburg, Russia Petersburg Nuclear Physics Institute named by B.P. Konstantinov of National Research Centre “Kurchatov Institute”, Gatchina, Leningrad region, Russia; Pavlov First St. Petersburg State Medical University, St. Petersburg, Russia; Pavlov First St. Petersburg State Medical University, St. Petersburg, Russia; Petersburg Nuclear Physics Institute named by B.P. Konstantinov of National Research Centre “Kurchatov Institute”, Gatchina, Leningrad region, Russia; Pavlov First St. Petersburg State Medical University, St. Petersburg, Russia; Pavlov First St. Petersburg State Medical University, St. Petersburg, Russia; Pavlov First St. Petersburg State Medical University, St. Petersburg, Russia; Pavlov First St. Petersburg State Medical University, St. Petersburg, Russia; Pavlov First St. Petersburg State Medical University, St. Petersburg, Russia Petersburg Nuclear Physics Institute named by B.P. Konstantinov of National Research Centre “Kurchatov Institute”, Gatchina, Leningrad region, Russia; Pavlov First St. Petersburg State Medical University, St. Petersburg, Russia Petersburg Nuclear Physics Institute named by B.P. Konstantinov of National Research Centre “Kurchatov Institute”, Gatchina, Leningrad region, Russia

**Keywords:** familial hypercholesterolemia, dyslipidemia, targeted sequencing, genetic risk scale, reverse cholesterol transport, семейная гиперхолестеринемия, дислипидемия, таргетное секвенирование, шкала генетического риска, обратный транспорт холестерина

## Abstract

Cardiovascular diseases are the leading cause of death both in Russia and in the world. One of the factors predisposing to the development of cardiovascular diseases is lipid metabolism disorders (dyslipidemias), which contribute to the progression of atherosclerosis. Currently, there are known genes associated with the development of monogenic forms of lipid metabolism disorders characterized by marked changes in lipid levels. However, identifying individuals with an increased genetic risk of dyslipidemia remains an unsolved problem, due to the polygenic nature of most cases. The aim of this work was to study the spectrum of rare variants in the cholesterol transporter genes ABCA1, ABCG1, ABCG5, ABCG8 and NPC1L1 that occur in patients with lipid metabolism disorders in the population of the Northwestern region of Russia. The search for rare variants (gnomAD frequency less than 1 %) in the ABCA1, ABCG1, ABCG5, ABCG8 and NPC1L1 genes was performed using targeted sequencing data for 169 patients with lipid metabolism disorders. 14 variants were identified in the ABCA1 gene (17 patients); 4 variants, in the ABCG1 gene (5 patients); 11 variants, in the ABCG5 gene (18 patients); and 7 variants, in the ABCG8 gene (11 patients). The frequency of some of them, according to the RUSeq database, is higher than in the global population. 19 patients (11 %) were carriers of the p.(Val177Ile)/p.(His221Tyr)/p.(Ala271Phe) haplotype in the NPC1L1 gene, which may be specific to the Russian population, meaning that these variants are not rare, but polymorphic, and occur more frequently in patients with impaired lipid metabolism. Influence of the p.(Val177Ile) variant of the NPC1L1 gene on the development of atherosclerosis was assessed using additional sample sets (a group of patients with atherosclerosis, a control group), but no significant differences in genotype frequencies were revealed. Thus, at present, there are insufficient data to support the role of the p.(Val177Ile)/p.(His221Tyr)/p.(Ala271Phe) haplotype of the NPC1L1 gene in the development of dyslipidemia and atherosclerosis. The study draws attention to the population specificity of a number of variants in cholesterol transporter genes, in particular in the NPC1L1 gene, for the Northwestern region of Russia. The data can be further used for design and calculation of genetic risk scores for dyslipidemia.

## Abstract (ru)

Сердечно-сосудистые заболевания являются основной причиной смерти в России и мире. Один из предрасполагающих к развитию сердечно-сосудистых заболеваний факторов – нарушения липидного обмена (дислипидемии), способствующие прогрессированию атеросклероза. На настоящий момент известны гены, ассоциированные с развитием моногенных форм нарушений липидного обмена, характеризующихся выраженным изменением уровня липидов. Однако выявление лиц с повышенным генетическим риском развития дислипидемии остается нерешенной задачей, что связано с полигенной природой большинства случаев. Целью настоящей работы было изучить спектр редких вариантов в генах транспортеров холестерина ABCA1, ABCG1, ABCG5, ABCG8 и NPC1L1, которые встречаются у пациентов с нарушениями липидного обмена в популяции Северо-Западного региона России. Проведен поиск редких вариантов (частота gnomAD менее 1 %) в генах ABCA1, ABCG1, ABCG5, ABCG8 и NPC1L1 с использованием данных таргетного секвенирования для 169 пациентов с нарушениями липидного обмена. Выявлено 14 вариантов в гене ABCA1 (17 пациентов), 4 варианта в гене ABCG1 (5 пациентов), 11 вариантов в гене ABCG5 (18 пациентов) и 7 вариантов в гене ABCG8 (11 пациентов). Частота некоторых из них, согласно базе данных RUSeq, была выше, чем в общемировой популяции. 19 пациентов (11 %) были носителями гаплотипа p.(Val177Ile)/p.(His221Tyr)/p.(Ala271Phe) гена NPC1L1, который предположительно может быть специфичен для российской популяции, т. е. эти варианты являются не редкими, а полиморфными и встречаются чаще у пациентов с нарушениями липидного обмена. Для варианта p.(Val177Ile) гена NPC1L1 был проведен анализ его вклада в развитие атеросклероза с использованием дополнительных выборок (группа пациентов с атеросклерозом, контрольная группа), который не выявил достоверных различий в частотах генотипов. Таким образом, в настоящее время данных в пользу влияния гаплотипа p.(Val177Ile)/ p.(His221Tyr)/p.(Ala271Phe) гена NPC1L1 на развитие дислипидемии и атеросклероза недостаточно. Проведенное исследование заставляет обратить внимание на популяционную специфичность ряда вариантов в генах транспортеров холестерина, в частности в гене NPC1L1, для Северо-Западного региона России. Полученные данные в дальнейшем могут быть учтены в разработке шкал генетического риска развития дислипидемий.

## Introduction

Cardiovascular diseases (CVDs) are the leading cause of death in Russia and worldwide (Danilova et al., 2021; Heron, 2021). The multifactorial nature of CVDs underscores the importance of studying new markers of the pathological process initiation, including genetic ones. The contribution of the genetic component to the development of heart and vascular diseases is estimated at 40–50 % (McPherson, Tybjaerg‑Hansen, 2016; Roberts et al., 2021). Hypercholesterolemia, which often has a hereditary nature, promotes the formation of atherosclerotic plaques in the coronary vessels, which in turn leads to the developvent of coronary artery disease (CAD) and acute myocardial infarction (AMI) (Prasad, Mishra, 2022). Familial hypercholesterolemia (FH) occupies a special place among hereditary lipid metabolism disorders, as it significantly increases the risk of developing CVDs (Tokgozoglu, Kayikcioglu, 2021). We and other authors have shown that in most cases, FH is caused by pathogenic variants in the LDLR and APOB genes. However, depending on the severity of hypercholesterolemia, the genetic cause of the disease cannot be identified in 40–60 % of cases (Shakhtshneider et al., 2021; Miroshnikova et al., 2023a, 2025). Thus, discovering rare genetic variants, which, in combination, can have a cumulative effect on the development of polygenic cases of hypercholesterolemia and associated CVDs, remains relevant.

In a study by A.N. Meshkov and co‑authors, an increased risk of CAD in carriers of rare and low‑frequency variants in genes associated with lipid metabolism disorders was demonstrated (Meshkov et al., 2022). Rare functional genetic variants associated with cholesterol metabolism were found in 60 % of patients with AMI (Pan‑Lizcano et al., 2022). It has been shown that 25 % of loci associated with AMI belong to lipid metabolism genes (Musunuru, Kathiresan, 2016). Rare genetic variants have a population prevalence of <1 % and may not be statistically associated with diseases of interest in large sample sets. However, even a small increase in allele frequency (1–5 %) in patients may indicate its influence on complex diseases and traits (Cross et al., 2022; Li et al., 2024). Moreover, such genetic variants may be population‑specific and should be taken into account during development of genetic risk scores for a specific population.

Atherogenic imbalance of blood plasma lipid fractions manifests as an increase in the concentration of total cholesterol (TC), low‑density lipoprotein cholesterol (LDL‑C), and a decrease in high‑density lipoprotein cholesterol (HDL‑C). Transmembrane cholesterol transporters play an important role in regulation of cellular cholesterol levels, in the formation of lipoprotein particles, intestinal cholesterol absorption, and cholesterol excretion from the body (Yu, Tang, 2022). ATP‑binding cassette (ABC) transporters – ABCA1 and ABCG1 – transport cholesterol to anti‑atherogenic high‑density lipoproteins (HDLs) (Yu, Tang, 2022). Mutations in the ABCA1 gene lead to the development of an autosomal recessive disorder – Tangier disease – characterized by an almost complete absence of HDLs in blood plasma and premature development of atherosclerosis (Koseki et al., 2021). Transporters ABCG5 and ABCG8 regulate the absorption of choles- terol and plant sterols in the intestine. Mutations in these genes lead to the development of sitosterolemia, which has clinical manifestations similar to FH (Tada et al., 2022). The NPC1L1 transporter, known as Niemann‑Pick C1‑like protein 1, is also involved in intestinal cholesterol absorption and is a target for the lipid‑lowering drug ezetimibe (Zhang et al., 2022). It can be hypothesized that combinations of rare variants in cholesterol transporter genes may contribute to the development of dyslipidemias and CVDs (Ghaleb et al., 2022; Meshkov et al., 2022).

The aim of this study was to investigate the spectrum of rare variants in the cholesterol transporter genes ABCA1, ABCG1, ABCG5, ABCG8, and NPC1L1 that occur in patients with lipid metabolism disorders in the population of the Northwestern region of Russia.

## Materials and methods

The study was approved by the Local Ethics Committee of the Pavlov First Saint Petersburg State Medical University of the Ministry of Health of the Russian Federation (Protocol No. 275 dated September 4, 2023). All participants provided written informed consent.

**Fig. 1.**
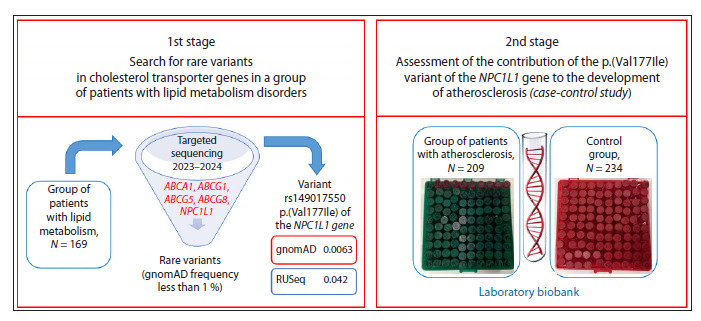
Research design

The overall study design is presented in Figure 1.

**Sequencing data analysis**

The search for rare variants in the cholesterol transporter genes ABCA1, ABCG1, ABCG5, ABCG8 and NPC1L1 was carried out using NGS sequencing data obtained for 169 patients with lipid metabolism disorders: 13 adults (aged 24 to 85 years, mean age 52.3 ± 14.6; 65 men and 69 women); 35 children (aged 3 to 17 years, mean age 9.7 ± 3.8; 18 boys and 17 girls). These patients were referred for genetic diagnosis of hereditary dyslipidemias to the Department of Molecular Genetic and Nanobiological Technologies from various clinical diagnostic centers in Saint Petersburg (including Pavlov First Saint Petersburg State Medical University) during 2023–2024.

Criteria for genetic testing prescription were as follows: 1) possible/probable/definite FH in patients over 18 years of age in accordance with Dutch diagnostic criteria (Kukharchuk et al., 2020); 2) probable/definite FH in patients under 18 years of age in accordance with Simon Broome criteria (Yezhov et al., 2019); 3) pronounced hypertriglyceridemia (individuals with triglyceride concentrations from 3.3 to 10.5 mmol/L were included).

Libraries were prepared using a set of Prep&Seq reagents (Parseq Lab Co, Russia) and a custom panel “Dyslipidemia and CVD risk”, including coding regions of the following genes: ABCA1, ABCG1, ABCG5, ABCG8, ANGPTL3, APOA1, APOA4, APOA5, APOB, APOC2, APOC3, APOE, CETP, CREB3L3, GCK, CYP27A1, CYP7A1, GPD1, GPIHBP1, HNF1A, LCAT, LDLR, LDLRAP1, LIPA, LIPC, LIPG, LMF1, LPL, LRP6, MTTP, MYLIP, NPC1L1, PCSK9, PNPLA5, SAR1B, SCARB1, SORT1, STAP1 and TTR (VariFind LM assay IL-v1.1.1, Parseq Lab Co, Russia). Sequencing was performed on a MiSeq instrument (Illumina, Inc., USA). The sequencing data were processed using the automated Seq<Go Software (Parseq Lab Co, Russia). Identified genetic variants were annotated and described according to the guidelines of the Human Genome Variation Society (HGVS) (www.hgvs.org) and presented in tabular format. Next, we selected rare variants (gnomAD frequency less than 1 %) in the cholesterol transporter genes ABCA1, ABCG1, ABCG5, ABCG8 and NPC1L1. The frequencies of the selected variants were compared with the Russian database of genetic information RUSeq (http:// ruseq.ru/#/) (Barbitoff et al., 2024). The OMIM (https://omim. org/), gnomAD (https://gnomad.broadinstitute.org/), ClinVar (https://www.ncbi.nlm.nih.gov/clinvar/), HGMD (https:// www.hgmd.cf.ac.uk/ac/index.php), LOVD (https://www. lovd.nl/) databases and literature data were used to assess the clinical relevance of the identified nucleotide sequence variants. The assessment of the clinical significance (pathogenicity) of the identified variants was carried out on the basis of Russian recommendations for the interpretation of data obtained by mass parallel sequencing methods, as well as the recommendations of ACMG2015 (Ryzhkova et al., 2019).

The verification of the p.(Val177Ile), p.(His221Tyr) and p.(Ala271Phe) variants of the NPC1L1 gene, which were considered to compose a haplotype, was carried out by Sanger sequencing on a Nanophor-05 sequencer (Syntol, Russia) using the BigDye™ Terminator v3.1 Cycle Sequencing Kit (Applied Biosystems, USA). The results were analyzed using the Mutation Surveyor software (Soft Genetics, USA). The primers for sequencing were selected using the online program Primer-BLAST (https://www.ncbi.nlm.nih.gov/tools/primerblast/) (Table 1).

**Table 1.**
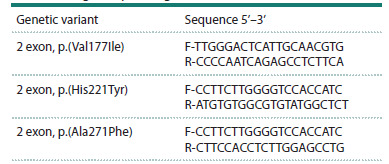
The nucleotide sequences of the primers used for Sanger sequencing

**Evaluating the contribution of the p.(Val177Ile) variant of the NPC1L1 gene to the development of atherosclerosis**

Characteristics of the groups. Genotyping of the p.(Val177Ile) variant of the NPC1L1 gene was performed for a total of 443 patients who were examined or treated at different times at Pavlov First Saint Petersburg State Medical University and whose DNA was available from the biobank of the Department of Molecular Genetic and Nanobiological Technologies: 1) patients with atherosclerosis of various localization (N = 209), 2) control group (N = 234).

The group of patients with early atherosclerosis included 209 patients (146 (70 %) men and 63 (30 %) women; average age 54.6 ± 8.5 years; the average age of the first clinical manifestations was 48.2 ± 6.8 years) with atherosclerosis of the arteries of various localization (coronary, cerebral, lower limb arteries), confirmed by angiographic examination (Table 2). The selection criterion was the presence of hemodynamically significant stenoses in at least one artery of one of the three main arterial basins – the cerebral, coronary, and lower extremities. It should be noted as a limitation that the sample set was formed based on the principle of confirmed atherosclerosis, regardless of whether patients had dyslipidemia, since the duration and effectiveness of statin treatment in most cases was difficult to evaluate.

**Table 2.**
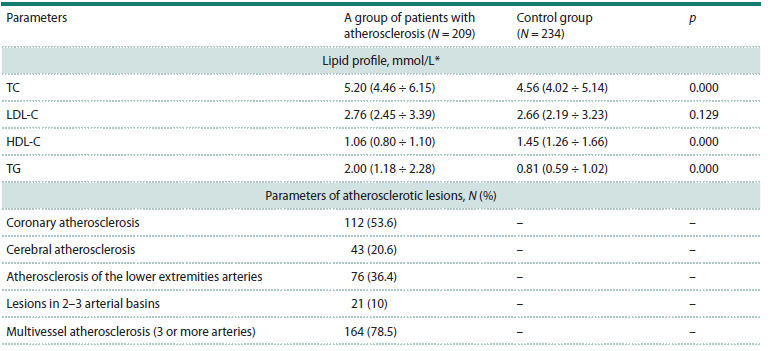
Clinical and biochemical characteristics of the studied groups Note. TC – total cholesterol, LDL-C – cholesterol of low-density lipoproteins, HDL-C – cholesterol of high-density lipoproteins, TG – triglycerides. * The median and interquartile range (IQR) are indicated.

The control group included 234 individuals (154 (66 %) men and 80 (34 %) women; average age 48.3 ± 5.8 years – the age corresponded to the average age of onset of the disease in patients with atherosclerosis) without lipid metabolism disorders, obesity and cardiovascular diseases. Control group individuals were examined by a cardiologist, and the examinations performed included electrocardiography, bicycle ergometry, and echocardiography.

Identification of the p.(Val177Ile) variant of the NPC1L1 gene using polymerase chain reaction and restriction analysis. Genotyping of the rs149017550 chr7:44539868C>T c.529G>A p.(Val177Ile) variant of the NPC1L1 gene was performed by polymerase chain reaction (PCR) and subsequent restriction analysis. The primers for PCR were selected using the online program Primer-BLAST (https://www.ncbi.nlm. nih.gov/tools/primer-blast/). The nucleotide sequence of the primers was (5′–3′): forward – TTGGGACTCATTGCAAC GTG, reverse – CCCCAATCAGAGCCTCTTCA. As a result of amplification with these primers, a PCR product with a size of 352 bp was obtained.

For restriction analysis, the PCR product was incubated with 1 unit of Fok I endonuclease in buffer Y containing: 33 mmol Tris acetate (pH 7.9 at 25 °C), 10 mmol magnesium acetate, 66 mmol potassium acetate, 1 mmol DTT, at +37 °C overnight. The results were visualized using polyacrylamide gel electrophoresis: depending on the genotype, DNA fragments of 352, 224, and 128 bp in length were obtained (for GG, only a fragment of 352 bp; for GA, fragments of 352, 224, and 128 bp; for AA, fragments of 224 and 128 bp).

**Statistical analysis**

The statistical analysis was performed using the SPSS version 17.0 software. The chi-square test was used to compare categorical variables. The correspondence of the data to the normal distribution was checked using the Kolmogorov– Smirnov test. The nonparametric Mann–Whitney test was used to compare quantitative indicators between two independent groups (patient-control). The assessment of the possible influence of the NPC1L1 genotype on the development of atherosclerosis, adjusted for gender and age, was performed using multifactorial logistic regression analysis.

## Results

**Analysis of the prevalence of rare variants in the ABCA1, ABCG1, ABCG5, ABCG8 and NPC1L1 genes in patients with lipid metabolism disorders**

We analyzed targeted sequencing data for 169 patients with hyperlipidemia, specifically searching for rare variants (gnomAD frequency <1 %) in the cholesterol transporter genes – ABCA1, ABCG1, ABCG5, ABCG8 and NPC1L1. The results are presented in Tables 3–7.

14 variants in the ABCA1 gene were identified in 17 patients, and four variants in the ABCG1 gene were found in five patients (Tables 3 and 4). All identified variants are classified as benign in public databases. The ABCA1 transporter is an important regulator of HDL biogenesis, mediating the transfer of cholesterol from cells to nascent pre‑beta‑HDL particles. Therefore, ABCA1 gene variants affecting protein function may be associated with reduced plasma HDL‑C levels, contributing to the development of atherogenic dyslipidemia (Shim et al., 2021). A female patient with a clinical presentation of FH and low HDL‑C levels (1.2 mmol/L, below the lower normal limit for women) was a carrier of two rare ABCA1 variants: rs187652566 p.(Cys887Phe) and rs138422574 p.(Val1674Ile). It is noteworthy that the frequencies of two ABCA1 variants in the Russian population are higher than the gnomAD frequencies: rs764824326 p.(Glu450Lys) and rs138422574 p.(Val1674Ile) (Table 3)

**Table 3.**
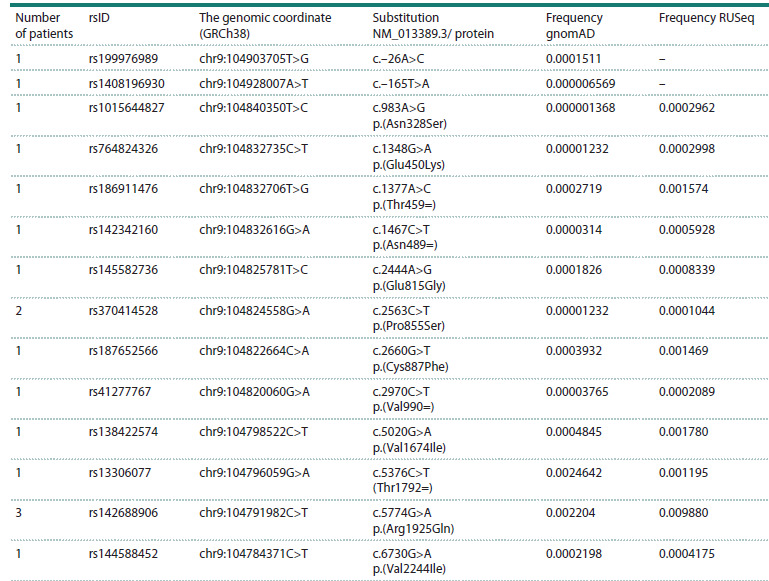
ABCA1 rare variants identified in patients with lipid metabolism disorders Note. All variants are likely benign.

**Table 4.**
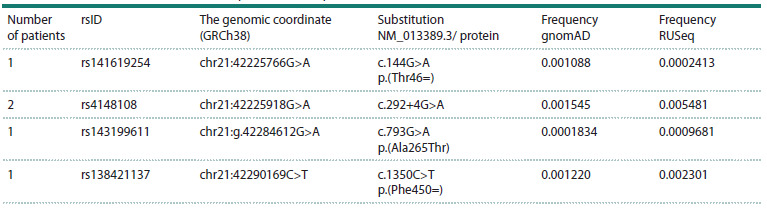
ABCG1 rare variants identified in patients with lipid metabolism disorders Note. All variants are likely benign.

The ABCG1 transporter provides saturation of mature HDL cholesterol for subsequent transport to the liver. According to the RUSeq database, two variants of the ABCG1 gene are more common in the Russian population: rs4148108 (c.292+4G>A) and rs143199611 p.(Ala265Thr) (Table 4).

11 variants were identified in the ABCG5 gene in 18 patients, and seven variants in the ABCG8 gene were found in 11 patients, including two pathogenic variants (ABCG8 rs137852987 p.(Trp361Ter) in three and ABCG8 rs769576789 p.(Leu572Pro) in two patients) and nine variants of uncertain significance (VUS) (Tables 5–6). Thus, pathogenic variants in the ABCG8 gene were identified in five patients (3 %). Variants of uncertain clinical significance, rs141828689 p.(Arg198Gln) and rs145164937 p.(Ala98Gly) in the ABCG5 gene, previously described in patients with FH (Totoń-Żuranska et al., 2023), were identified in this study in two patients with hypercholesterolemia and early-onset CVD; the frequency of these genetic variants is higher in the Russian population according to the RUSeq database (Table 5). However, there is currently insufficient data to classify these variants as pathogenic. In addition, our study identified variants of uncertain clinical significance: rs1167870780 p.(Leu195Gln), rs776335488 p.(Ser569Pro) and rs113005049 p.(Ala642Thr). Variants rs1167870780 p.(Leu195Gln) and rs776335488 p.(Ser569Pro) were previously described in patients with sitosterolemia (Meašić et al., 2021; Chubykina et al., 2025), variant rs113005049 p.(Ala642Thr) – in a patient with FH (Averina et al., 2018). Variants in the ABCG8 gene, rs776335488 p.(Ser569Pro), rs189249032 p.(Tyr613His) and rs113005049 p.(Ala642Thr), as well as the pathogenic variant rs1167870780 p.(Leu572Pro), are found in the Russian population more often than it is indicated in the gnomAD database (Table 6).

**Table 5.**
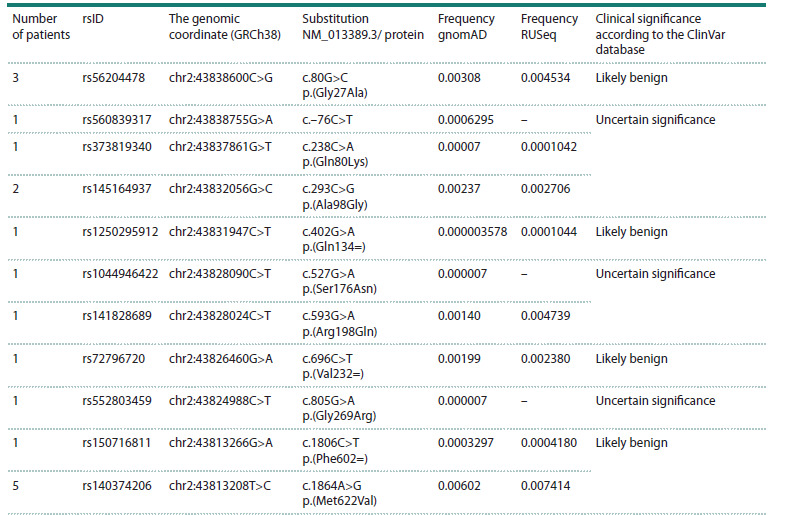
ABCG5 rare variants identified in patients with lipid metabolism disorders

**Table 6.**
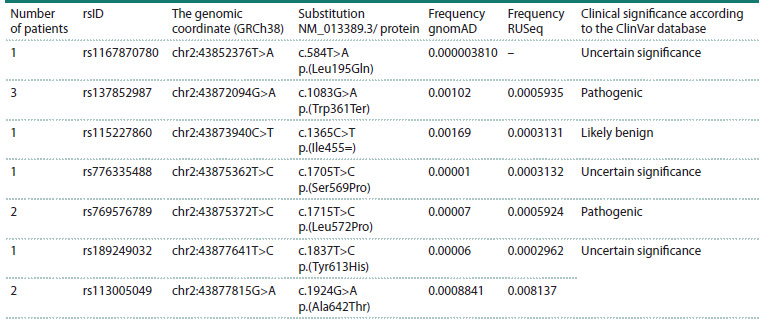
ABCG8 rare variants identified in patients with lipid metabolism disorders

**Fig. 2.**
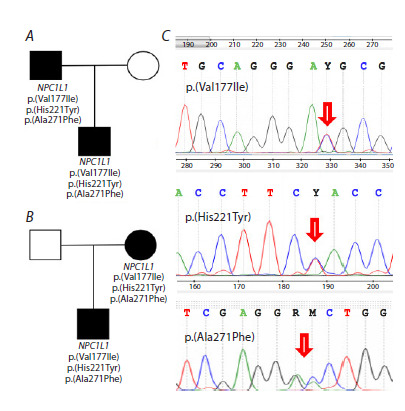
Sanger sequencing for two familial cases of haplotype p.(Val177Ile)/p.(His221Tyr)/p.(Ala271Phe) in the NPC1L1 gene. A, B – pedigrees of patients carrying the p.(Val177Ile)/p.(His221Tyr)/ p.(Ala271Phe) haplotype of the NPC1L1 gene; C – example of Sanger verification of the p.(Val177Ile), p.(His221Tyr) and p.(Ala271Phe) variants of the NPC1L1 gene

**Fig. 3.**
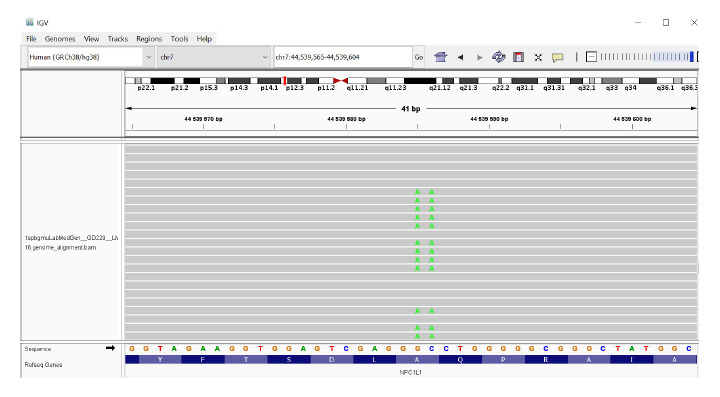
Results of NGS-sequencing for the p.(Ala271Phe) variant of the NPC1L1 gene (bam-file).

Nine NPC1L1 rare variants were identified in 29 patients (Table 7). 19 patients were carriers of three variants – p.(Val177Ile), p.(His221Tyr) and p.(Ala271Phe) – which, as we assume, are in linkage disequilibrium and in combination compose a haplotype (Fath et al., 2020) with an increased frequency in the Russian population (Table 7). This assumption is supported by the results of Sanger sequencing performed in two families, where relatives of the patients were available for analysis (Fig. 2). We also identified a homozygous carrier of the NPC1L1 p.(Val177Ile)/p.(His221Tyr)/p.(Ala271Phe) haplotype. Note that the single‑nucleotide substitutions described in the database – rs117724326 p.(Ala271Val) (frequency according to RUSeq: 0.04729) and rs139533378 p.(Ala271Ser) (frequency according to RUSeq: 0.04729) – in adjacent nucleotides are in fact a single variant, c.811_812delinsTT p.(Ala271Phe) (Fig. 3). It should be noted that the NPC1L1 p.(Val177Ile)/p.(His221Tyr)/p.(Ala271Phe) haplotype is typically found in patients in whom monogenic dyslipidemia cannot be confirmed. In our study, only 1 carrier of this haplotype also had a mutation in the LDLR gene.

**Table 7.**
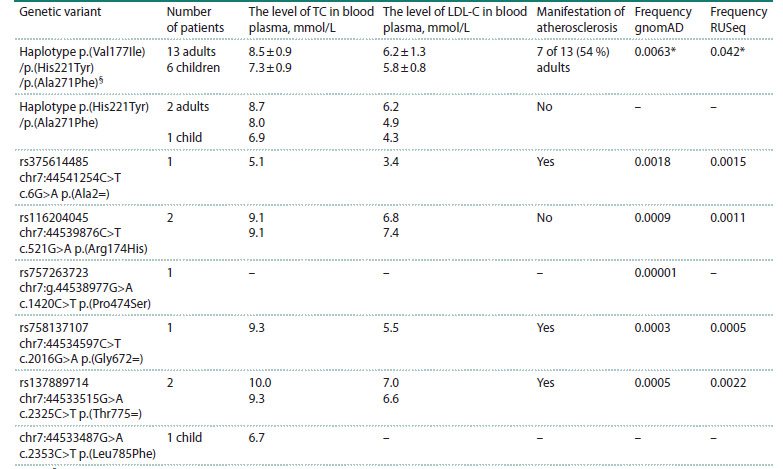
NPC1L1 rare variants identified in patients with lipid metabolism disorders Note. § Combination of genetic variants: chr7:44539868C>T c.529G>A p.(Val177Ile) (rs149017550), chr7:44539736G>A c.661C>T p.(His221Tyr) (rs114376659) and chr7:44539585_44539586GC>AA c.811_812delinsTT p.(Ala271Phe) (rs117724326/rs139533378). * TC – total cholesterol; LDL-C – low-density lipoprotein cholesterol. The frequency is stated for rs149017550 сhr7: 44539868C>T c.529G>A p.(Val177Ile).

**Table 8.**
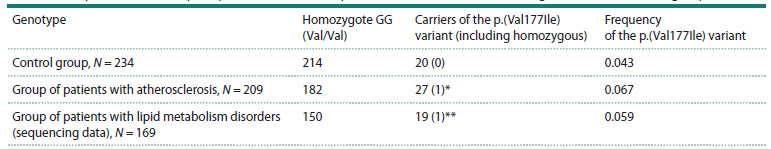
Comparison of the frequency of the rs149017550 p.(Val177Ile) variant of the NPC1L1 gene across all studied groups * p = 0.136 when compared with the control group; the groups are comparable in age and gender. ** p = 0.366 when compared with the control group; the groups are not comparable in age and gender

**Evaluation of the contribution of the p.(Val177Ile) variant of NPC1L1 gene to the development of atherosclerosis**

The NPC1L1 gene variants p.(Val177Ile), p.(His221Tyr) and p.(Ala271Phe) are located in the N‑terminal domain of the NPC1L1 protein, which is responsible for cholesterol binding (Hu et al., 2021). Carriage of this haplotype may lead to enhanced intestinal cholesterol absorption and may be associated with the development of dyslipidemia and arterial atherosclerosis (Fath et al., 2020). A method based on PCR and restriction analysis was developed for the identification of the p.(Val177Ile) variant of the NPC1L1 gene. Genotyping was performed in a group of patients with atherosclerosis of various localizations, as well as in a control group (Table 8)

The frequency of the p.(Val177Ile) variant of the NPC1L1 gene in the control group was 0.043, what is comparable with the frequency reported in the RUSeq database (0.042). In the group of patients with atherosclerosis, the frequency was 0.067, which is higher than in the control group; however, the differences did not reach statistical significance. When performing regression analysis using the entire dataset – and additionally accounting for factors such as sex, age, and the presence of atherosclerosis – we were unable to demonstrate an association between the p.(Val177Ile) variant of the NPC1L1 gene and the development of atherosclerosis on the base on dyslipidemia. This result may be attributed to the insufficient size of the study sample sets; further research is required.

## Discussion

Due to the high prevalence of CVDs, the impact of various factors on the development of this pathological process remains a relevant issue. A significant genetic contribution to the progression of CVDs highlights the prospects of clarifying the status of rare variants associated with lipid accumulation and subsequent vascular damage (Meshkov et al., 2022). The complexity of the genetic architecture of dyslipidemias underscores the importance of implementing modern diagnostic approaches for screening and early identification of carriers of rare genetic variants (Kalwick, Roth, 2025).

The most common hereditary dyslipidemia is FH (Miroshnikova et al., 2023a). FH is an autosomal dominant genetic disorder characterized by high levels of cholesterol and LDL‑C in blood plasma. Typical phenotypic manifestations include xanthomas, xanthelasmas and corneal arcus. Genetic diagnosis in patients with FH can be discovered in only 40–60 % of cases with a monogenic nature of the disease (Medeiros et al., 2024; Miroshnikova et al., 2025). The prevalence of FH varies from 1:250 to 1:173 depending on the population (Meshkov et al., 2021; Toft‑Nielsen et al., 2022). Additionally, there are more rare monogenic dyslipidemias, such as lipoprotein lipase deficiency (Hegele et al., 2015), dysbetalipoproteinemia (Heidemann et al., 2022), sitosterolemia (Miroshnikova et al., 2023b), and others (Ivanova et al., 2020). The remaining cases of lipid metabolism disorders may be of polygenic nature (Futema et al., 2015).

As previously shown in GWAS studies, common genetic variants play an important role in predisposing to lipid metabolism disorders (Ripatti et al., 2016), but rare and low‑frequency variants may also significantly contribute to the development of dyslipidemia and subsequent progression of atherosclerosis (Hindy et al., 2022). The population‑specific distribution of genetic variants highlights the need to assess allele frequencies separately for each population (Read et al., 2021; Senftleber et al., 2024; Fan et al., 2025). In this study, we examined the spectrum of rare variants (gnomAD frequency <1 %) in cholesterol transporter genes – ABCA1, ABCG1, ABCG5, ABCG8 and NPC1L1 – in patients with lipid metabolism disorders in the population of the Northwestern region of Russia.

We identified several variants in the ABCG5 and ABCG8 genes previously reported in patients with FH or sitosterolemia (ABCG5 rs141828689 (p.Arg198Gln); ABCG8 rs776335488 p.(Ser569Pro), rs769576789 p.(Leu572Pro), rs189249032 p.(Tyr613His), rs113005049 p.(Ala642Thr)), several among them were more prevalent in the Russian population (Tables 5 and 6). Homozygous and compound heterozygous pathogenic variants in these genes cause a rare autosomal recessive genetic disorder, sitosterolemia, with an estimated prevalence of 1:200,000 (Pshenichnikova et al., 2024). This condition is characterized by elevated blood levels of plant sterols, as well as total cholesterol and LDL‑C. Patients with sitosterolemia often present with xanthomas and early‑onset cardiovascular diseases, making the clinic signs similar to FH. Heterozygous carriage of pathogenic ABCG5 or ABCG8 variants does not cause sitosterolemia but may increase the risk of hypercholesterolemia (Reeskamp et al., 2020). In our study, heterozygous carriers of pathogenic and likely pathogenic variants in ABCG5 and ABCG8 accounted for 3 %, which is consistent with previously published data (Reeskamp et al., 2020; Medeiros et al., 2024).

Our study allowed us to pay attention to the increased frequency of the p.(Val177Ile)/p.(His221Tyr)/p.(Ala271Phe) haplotype of the NPC1L1 gene both in patients with lipid metabolism disorders in the Northwestern region of Russia and in the Russian population overall. Active investigation of the NPC1L1 transporter is driven by the potential for dyslipidemia therapy. Currently, ezetimibe – an NPC1L1 inhibitor that blocks intestinal cholesterol absorption – is widely used. Ezetimibe acts as an allosteric inhibitor of NPC1L1, inducing a “closed” conformation of the transporter, which disrupts cholesterol binding (Valdivia et al., 2023). Polymorphic variants in the NPC1L1 gene have been shown to account for inter‑individual differences in sensitivity to ezetimibe (Liao et al., 2022) and may lead to a complete lack of response (Mauriello et al., 2023). Therefore, studying NPC1L1 genetic variants as risk factors for dyslipidemia and as pharmacogenetic markers for predicting the efficacy of lipid‑lowering therapy is an important goal (Liao et al., 2022). Typically, variants in this gene are associated with reduced protein activity and are therefore protective, but variants that increase cholesterol absorption are also known – for example, p.(Arg174His), which we identified in two patients (Mokhtar et al., 2022).

Substitutions p.(Arg174His), p.(Val177Ile), p.(His221Tyr), and p.(Ala271Phe) are located in the N‑terminal domain of the NPC1L1 protein, which plays a key role in cholesterol uptake (Valdivia et al., 2023; Yoon et al., 2023). Variability in cholesterol absorption and plasma LDL‑C levels has been previously demonstrated to depend on a combination of rare variants in the NPC1L1 gene (Simonen et al., 2023). In the study by F. Fath et al., the NPC1L1 p.(Val177Ile), p.(His221Tyr) and p.(Ala271Phe) variants were described in a patient with hypercholesterolemia, and their combination was classified by the authors as a possible cause of the disease (Fath et al., 2020), which is consistent with our findings. For the Russian population, the p.(Val177Ile)/p.(His221Tyr)/p.(Ala271Phe) haplotype has been shown to be population‑specific: it is not rare but rather polymorphic. We also assessed for the first time the contribution of one of the haplotype variants, p.(Val177Ile), to atherosclerosis development. However, differences in genotype frequencies between patients with atherosclerosis and the control group did not reach statistical significance, which does not allow us to draw a conclusion about the impact of the p.(Val177Ile) variant in the NPC1L1 gene on atherosclerosis. Our results do not indicate a definitive absence of contribution from either the individual variant or the p.(Val177Ile)/p.(His221Tyr)/p.(Ala271Phe) haplotype to the polygenic background; thus, expanding the sample size in future studies may allow a more precise verification of the significance of these variants. Further research is needed to investigate the contribution of both individual variants and the haplotype to the polygenic risk of hypercholesterolemia and the efficacy of ezetimibe treatment.‑

## Conclusion

Currently, in the absence of monogenic variants, polygenic hypercholesterolemia is assumed: when no significant pathogenic genetic variants are present, the accumulation of common and rare genetic variants with small effects may lead to dyslipidemia. This study examined the spectrum of rare variants in cholesterol transporter genes – ABCA1, ABCG1, ABCG5, ABCG8 and NPC1L1 – in patients with lipid metabolism disorders who underwent genetic testing. Our work indicates a higher frequency of some rare variants in these genes – particularly NPC1L1 – in the Russian population. However, to determine their contribution to CVD risk, their prevalence must be assessed in larger patient groups. Additional studies are needed to develop population‑specific genetic risk scores that account for the cumulative contribution of risk and protective alleles in lipid metabolism‑related genes and can be used to predict individual risk of dyslipidemia and cardiovascular diseases.

Study limitations. The limitations of the study include: relatively small sample sizes for investigating population‑specific patterns of genetic variant distribution; specific features of sample formation. In particular, the first sample set included individuals with severe dyslipidemia who were prescribed genetic testing. Consequently, the proportion of individuals with monogenic forms of dyslipidemia – primarily familial hypercholesterolemia (FH) – in this sample set was relatively high, accounting for 30 %. On the other hand, it should be noted that the haplotype p.(Val177Ile)/p.(His221Tyr)/p.(Ala271Phe) of the NPC1L1 gene was detected predominantly in individuals without an identified monogenic cause of dyslipidemia, with the exception of one patient. A limitation of the sample set used in the second stage of the study is that the group of patients with atherosclerosis was formed based on the presence of the disease, regardless of whether the patients initially had dyslipidemia. This is because the duration and effectiveness of statin treatment were difficult to track in most cases.

## Conflict of interest

The authors declare no conflict of interest.
